# Hospital and Institutional News

**Published:** 1918-06-08

**Authors:** 


### HOSPITAL AND INSTITUTIONAL NEWS.

### AMERICAN WOUNDED AT WINDSOR.

THE King has given permission for a hut hospital to be built, according to the latest design, in Windsor Great Park for the benefit of American wounded, who are to be brought to England for treatment. A fully equipped hospital of 500 beds is the splendid gift which the Joint War Committee of the British Red Cross Society and the Order of St. John has decided to present to the American Red Cross. The site of the new hospital has a fine view of Windsor Castle, and it is hoped that the institution will be ready early in the autumn. The gift has been accepted by Major Endicott on behalf of the American Red Cross. The King's action in granting a site in Windsor Park cannot fail to be appreciated in America, and by the gallant men whom she is sending to France.

### THE NEW EXTENSIONS AT LEEDS.

FIVE new operating theatres and their annexes have been opened informally at Leeds General Infirmary. They form part of the King Edward Memorial extension. Four of the new theatres are to be used for general operations. The fifth is to be devoted to eye, ear, throat, and nose cases. In place of the wooden gallery in which students used to be accommodated the new theatres are provided with a raised platform. The General Infirmary at present accommodates 250 wounded soldiers, and the operations have numbered twenty a day on an average, and sometimes many more. The two theatres on which the hospital has been depending have, therefore, been overstrained by the amount of work. The new theatres, placed in one of the new wings, are directly above the out-patient department, which is now in use, although not wholly complete. Thus in a quiet way this great extension scheme proceeds, and the great task which the hospital authorities undertook in difficult circumstances is being steadily accomplished.

### ANOTHER HOSPITAL AMALGAMATION?

THE fate of the Royal Albert Hospital, Devonport, so often threatened with closure, seems really in the balance at last. It has long suffered from want of support. In February an appeal was issued for £12,500. This has produced less than £2,000. A meeting of governors has been held, at which there was a dearth of constructive proposals till one speaker aptly reminded those present that they had met to devise means for keeping the hospital open, not to close it. The old proposal to amalgamate with the South Devon and East Cornwall Hospital was again raised, though the latter has previously given little encouragement to it. It was finally decided to adjourn the meeting for three months. This seems to show that the governors have not the strength of mind to come to any decision. We put this baldly, because one suggestion was made by the acting honorary secretary, Mr. F. H. Gertie, who had a definite policy of his own. This was to appoint an energetic paid secretary, charged with three duties. The first was to secure new annual subscribers, of whom there are now only 240. The second, to form working men's organisations on the lines of that started in H.M.'s Dockyard, which has guaranteed to raise £1,000 a year. The third was to invite all patients to contribute a thankoffering according to their means. These proposals were never deliberated. They were never even moved! Yet it is largely due to Mr. Gertie, as the treasurer acknowledged, that the recent effort was made. After all, it did raise nearly £2,000, which shows that he is the man to follow. One or two speakers thought well of his policy, but no one took it up. Why must three months be wasted before doing so? Such inertia invites the disaster which it does not dare to face.

### THE BOMBING OF HOSPITALS.

THE following letter, dated the 31st ultimo, signed "Englishman," was published in the *Times* of June 4, under the above heading: "The wilful bombing of hospitals by the Germans is stirring the hearts of all right-thinking people to the very depths. If the Government can devise no other mode of reprisal it should at once intern, if necessary in cages, say 100 German prisoners in each hospital, employ them in menial duties, and give notice at once to enemy authorities; but let there be no delay." We should be glad to have the opinion of hospital managers and authorities on this suggestion, with any practical alternatives which may suggest themselves.

### BIRTHDAY HONOURS.

THE following names appear in the Honours List issued on H.M. the King's Birthday, June 3: —*K.C.B.* (*Civil Division*): Surgeon-General Humphry Davy Rolleston, C.B., M.D., F.R.C.P., R.N. *C.B.* (*Military Division*): Surgeon-General Patrick Brodie Handyside, M.D., R.N. *C.M.G.*: Mr. Henry Lindo Ferguson, M.D., Professor of Ophthalmology, and Dean of the Medical Faculty, University of Otago; Fleet-Surgeons Percy William Bassett-Smith, R.N., C.B.; Frederick J. Abercrombie Dalton, R.N.; Edward Sutton, R.N.; David Walker Hewitt, R.N., M.B.; William Wallace Keir, R.N., M.B. *C.I.E.*: Lieut.-Col. Herbert Austen Smith, I.M.S.; Major and Brevet Lieut.-Col. Frederick Adolphus Fleming Barnardo, M.B., F.R.C.S.E., I.M.S.; Major Davis Heron, M.B., F.R.C.S.E., I.M.S.; and Col. Patrick Hehir, C.B., C.M.G., I.M.S. *G.C.V.O.*: Lieut.-General Sir Alfred Keogh, G.C.B., C.H. *K.C.V.O.*: Sir Alan Reeve Manby, M.V.O., and Lieut.-Col. Sir Edward Scott Worthington, C.M.G., M.V.O., R.A.M.C. *M.V.O.* (*Fourth Class*): Mr. Charles Percival White, M.B. *Order of the Companion of Honour*: Sir Frederick Treves, Bart., G.C.V.O., C.B.; Col. Sir H. C. Perrott, Bart., C.B., Vice-Chairman of the Joint War Committee of the Order of St. John and the British Red Cross Society. *I.S.O.*: Mr. William John Henderson Sinclair, M.B.; Senior

Assistant Surgeon and Hon. Major Michael Courtney, Indian Subordinate Medical Department. *Baronet*: Mr. William James Peake Mason, J.P., Chairman of the Executive of the British Ambulance Committee. *Knights*: Mr. Harry Baldwin, M.R.C.S.; Mr. John Reid, J.P., D.L., Director of Glasgow Royal Infirmary, Vice-President of the Princess Louise Scottish Hospital for Limbless Soldiers and Sailors; Col. Mayo Robson, C.V.O., F.R.C.S.; Mr. Peter Wyatt Squire (chemist to the Royal Family for fifty years); Mr. H. Warren, J.P. (Mayor of Poplar), Chairman and Treasurer of the War Hospital Supply Depôt, and founder of a voluntary fund for alleviating distress caused by air-raids. There is a long list of Army honours for services on the Western Front, Egypt, Italy, Salonika and elsewhere, which are to be published in seven sections, of which only three have appeared up to the day we go to press. We regret that pressure on our space prevents our mention of the names of the medical men who have been thus honoured.

### KING GEORGE'S FUND FOR SAILORS.

Captain A. W. Clarke, Deputy-Chairman of King George's Fund for Sailors, stated at the recent meeting of the General Council at Trinity House that the fund now amounted to £350,000. The General Council approved the draft of the Royal Charter of Incorporation and the statutes, which had been prepared by a special sub-committee, with the help of the honorary counsel, Mr. W. F. Hamilton, K.C., and Sir Perceval Nairne, honorary solicitor. These are now being submitted to the Home Office. Sir Richard Williams-Bulkeley said that a large measure of support would result from the appeal which the Duke of Connaught had made to India, the Dominions, and the Crown Colonies. Further support will be required, however, and the committee of Lloyd's Register on the day of the meeting voted £3,000 to the fund. Mr. Hartington Ratcliffe, R.N., Naval Representative at the Ministry of Pensions, and Mr. Thomas Spencer, consulting marine engineer, have been elected members of the Council.

### THE AFTER-TREATMENT OF WOUNDED.

The following official statement is worth recording. In reply to Major Astor, who stated that soldiers discharged from military and auxiliary hospitals were unable to obtain immediate admission to civilian hospitals for the further treatment which they required, Sir A. G. Boscawen has written: "Steps are being taken to eliminate so far as possible delay in obtaining immediate treatment for wounds and injuries. The War Office have agreed not to discharge men in future so long as there is any necessity for further operative treatment. In addition, arrangements have been concluded with that Department for the establishment of annexes for in- and out-patient treatment of discharged men in connection with military orthopædic centres. Where this is not possible annexes to civil hospitals will be provided. Arrangements for the admission of discharged men at special rates of payment have also been concluded with 353 hospitals throughout the kingdom."

### AN AMERICAN SURGEON ON IRELAND.

The Royal College of Surgeons of Ireland has conferred the honorary fellowship of the College upon Major Harvey Cushing, U.S.A. Medical Service, when the Lord-Lieutenant and Mr. Shortt were present. In acknowledging the honour, Major Cushing said that, after what might have seemed a long wait, America had realised that an insane nation was embroiling the world. Its ambition was unbelievable, and it was using unbelievable means to attain its end. There would be no half-measures, and America would stay in to the end. Canada was in, too, and they expected Ireland to be in it also. Major Cushing praised the gallantry of the Irish regiments, and hoped that a time was coming when a period of national service would help to smooth existing difficulties. It would be interesting to know how far these remarks reflect American opinion, and to what extent, if any, the acceptance of military service in America has modified American views of Ireland's attitude to the war.

### BACILLI AND BUSINESS.

It is always interesting to note the threads that join medical science and the business of the world. Anthrax is a disease that has shown some tendency of late to increase the number of its victims. Those who suffer are in chief part those concerned in the handling of the raw materials of our clothing. So many have the victims been that a Departmental Committee has been formed to report on ways to meet the danger. The Disinfection Sub-Committee has now reported on the results of its experiments, and from the report it appears that formaldehyde is the disinfectant capable of destroying the spores of anthrax without damaging the wools and other materials treated with it. Machinery and a process has been evolved whereby the antiseptic is brought in contact with the spores at a cost of between a penny and a halfpenny per pound of raw material, but of this charge on manufacture a part is saved, because the process of disinfection lessens the cost of other stages of preparation. Unfortunately all danger to the workers is not eliminated, for it is still necessary to handle the bales of fleece to open them to the process. It is, however, a great gain to make fewer the opportunities of infection. Sir William Middlebrook, M.P., is to be congratulated on the labours of his committee and his sub-committee.

### HOSPITAL VISITATION IN FRANCE.

From the commencement of the war the Salvation Army has organised visitation of the base hospitals in France. By permission of the authorities and in accordance with the hospital arrangements, the women officers go from cot to cot and render personal services to the helpless soldier. If he is too weak to write home the Salvationists perform the duty for him. Sometimes the men require various articles which they are unable to purchase. An Australian, for instance, wanted a watch, because he could not sleep at night, and wanted to know the time. He had no money, but said that his mother would pay back any advance. The gift of an illuminated-dial watch brought him great pleasure. In the case of men who die in France without the possibility of being visited by their friends a tribute of flowers is placed on their graves. A spray is taken, pressed, fixed on a card, and forwarded to relatives, who may never obtain the opportunity of seeing their man's last resting-place. The Salvation Army has created a special War Fund, which is distinct from the ordinary Fund, and is subject to Government audit. For this Flag Days are being organised throughout the country, and will take place in the metropolis on June 7-8.

### A NEW MECHANO-THERAPEUTICAL DEPARTMENT.

A new mechano-therapeutical department at the Royal Victoria and West Hants Hospital, Bournemouth, Boscombe Branch, has been opened by Mrs. Edward Douty, sister of Colonel Sir Gilbert Wills, Bart., one of the presidents of the hospital. The work has grown from quite a small beginning, until a formal opening ceremony was felt to be justified. The department, under the direction of Major A. H. Vernon, F.R.C.S., R.A.M.C. (T.), has proved of great benefit to large numbers of the military patients at the hospital. Two gifts were made at the opening ceremony, one of them, of £100, anonymously, and the other, of £50, a presentation sum to Mrs. Wyche in connection with her work at the Bournemouth War Supply Depôt, which she generously passed on to pay for the whirlpool bath, part of the equipment of the department. On the same occasion an interesting exhibition of needlework by the soldier patients in hospital attracted much attention during the afternoon.

### THE NEW AUXILIARY WAR HOSPITALS.

The War Office, which once frowned on privately organised hospitals, is asking for their establishment. For example, it has asked the Southwark division of the British Red Cross Society to form and equip such an auxiliary hospital, and the division has taken No. 119 Kennington Park Road, which will hold forty-two beds. In addition to the War Office allowance, it will cost £1,000 to adapt and equip the premises, and this sum has already been subscribed.

### WOMEN OUT-PATIENTS AND THE MINERAL WATER HOSPITAL, BATH.

Dr. Preston King, former Mayor of Bath, has raised the question of the expenses of the women out-patients of the Royal Mineral Water Hospital, which, he thinks, should be paid for by the hospital, because, were it not for the number of beds occupied by soldiers, they would be received as in-patients and avoid the expense of lodgings in the town. At a recent committee meeting Dr. King moved a resolution to this effect, and supported his contention by showing that the Stead Hostel and the G.F.S. Diocesan Lodge in Bath either had asked for or were in need of grants towards the expenses to which they were put in receiving these out-patients. In answer to the suggestion that the hospital could not afford the expense, Dr. King states that it is receiving from the Government £7,000 a year over and above its ordinary income, and that its total income last year was nearly £1,500 in excess of its expenditure. These arguments, which failed to gain a hearing at the committee meeting, have now appeared in the local newspapers, but the hospital's committee has returned no answer to them. We fear that there may be personal motives at work, but this should not prevent the case of these women being considered, nor the reasons why the committee is averse to assisting them or the institutions to which they go from being known. The reasons may be excellent, and, if so, it is a pity to withhold them. In Norfolk, where one hospital has received the patients of another to let the second admit military patients, the first has received a grant in return. What is the case against giving one at Bath?

### THE STANDARDISATION OF SALARIES.

An animated discussion lately took place at a meeting of the Westminster Board of Guardians, when the Hon. G. Wallop moved a resolution asking the Local Government Board to take steps to prevent competition between metropolitan Guardians in regard to employees. Four or five cases had been reported to him of officers having recently left the employment of the Westminster Board to enter that of other Unions who offered higher salaries. The mover of the resolution was vigorously opposed by Mr. W. A. Brinsdon, who said that the majority of Poor-Law officials were getting as little as it was possible to pay to educated people. He stated that he had seen an advertisement in that morning's newspapers offering £21 per annum for a probationer, whereas they in Westminster offered only £12. The debate was made interesting by the presence of Mr. Woolley Walden, chairman of the Metropolitan Asylums Board, who is an alderman of the Westminster City Council. Mr. Woolley Walden thought that it would be a bad thing to prevent competition, though he deprecated any attempt to draw officials from one authority to another by the offer of slightly higher salaries. Another Guardian suggested that in Westminster the stokers in their various institutions were paid varying wages. This home thrust led the supporters of the resolution to temporise, and in the end the board decided to ask their Finance Committee for a report on the wages of its own staff before approaching the Local Government Board on the vagaries of other bodies.

### THE REMUNERATION OF REGISTRARS.

Amongst the professional classes badly hit by the war are the Registrars of Births and Deaths, whose remuneration is dependent upon fees. In some cases it is stated that men who in pre-war times receive on the average £4 a week now barely get £3. The Local Government Board, as a temporary measure in such cases of hardship, would consider favourably, under the Local Authorities (Expenses) Act, 1887, the payment of gratuities by the Guardians within the scale laid down for war bonuses to Civil servants. The trouble, of course, arises through the registrars having to depend on fees and not having fixed salaries. In some unions the Guardians adopted the principle of paying their vaccination officers stated salaries, and abolishing the fee system, which has so far proved a success, and there is no reason why the same procedure could not be adopted with regard to registrars. Had the Local Government Board suggested such a procedure the difficulty would have been much easier to surmount.

### TREATMENT OF LONDON CONSUMPTIVES.

In a report presented to the London Insurance Committee it is recommended that papers relative to a proposed comprehensive scheme for the treatment of tuberculosis in London be sent to the President of the Local Government Board and the Chairman of the National Health Insurance Joint Committee, and that they be requested to take steps, in consultation with the London County Council and the London Insurance Committee, for the treatment of insured and uninsured persons. It is stated that the London County Council is willing to enter into an agreement with the Committee for providing a scheme, but is not prepared to erect institutions at present, or to join in a consulting committee. A lady member of the London Insurance Committee said that at a meeting on the 23rd ult. there was practically no waiting-list of soldiers. The figures for May 1, however, give 211 discharged sailors and soldiers on the waiting-list, as against 285 civilians. London consumptives have long needed something doing for them, and it is to be hoped appropriate measures will soon be taken.

### ASYLUM WORKERS' ASSOCIATION.

In connection with its annual meeting this year this Association has exhibited, in war-time, more energy and convincing results than ever before. No one can doubt that the Association is alive, or that it fulfils a useful purpose in a way which does honour to the memory of Honor Morten and all who are responsible for its management. Amongst them Dr. G. E. Shuttleworth occupies a prominent position, and his untiring energy and devotion are as noteworthy as they are entitled to national recognition. It is remarkable that the meeting should have been held at the Mansion House this year, one of the busiest years for a Lord Mayor of London ever faced by the holder of this ancient office. The speakers, too, included the Dean of Windsor and Dr. Charles Mercier, each of whom spoke with considerable force and interested the audience deeply. Dr. Mercier bore convincing testimony to the continued efficiency of our asylums, and contrasted the order, comforts, and treatment of the insane in the present day as compared with earlier periods. He mentioned again the fact that the keeper of George III., when asked how he managed his patient when he was tiresome, replied that he knocked him as flat as a flounder. Dr. Mercier's speech was a splendid testimony to the usefulness and value of the Asylum Workers' Association. Its recognition and support by the Lord Mayor will be grateful to all who are interested in efficient administration. We wish the Association increasing prosperity and the continuous extension of the many services it has and will render, in quietness truly, but with admirable energy and important results.

### ABERDARE HOSPITAL.

In recently proposing that the Merthyr Board of Guardians should renew their annual subscription of £25 to the Merthyr Hospital, which was temporarily withdrawn because one of the wards was closed and partly because of a dispute on workmen's representation, the Rector of Dowlais moved that a similar sum be voted to the recently opened general hospital at Aberdare. After some discussion this was agreed to. New hospitals do not always receive recognition of this kind.

**URGENT NOTICE.**

To ensure a copy of The Hospital regularly, readers must place a definite order with their Newsagent TO DAY. After the middle of June, in consequence of the new Paper Restrictions Order, it will not be possible for Newsagents to stock copies for chance customers.

DO NOT DELAY. ORDER

"THE HOSPITAL" TO-DAY

and obtain your copy regularly EVERY THURSDAY.

### THIS WEEK'S DRUG MARKET.

Business shows signs of improving. There is a fairly good undercurrent of inquiry which may lead to orders in due course. Makers of strychnine have again raised their quotations; this is the third advance within a comparatively short period. Other articles which are quoted higher include hydrastin, jalap resin, leptandrin, and apiol. Balsam copaiba continues scarce. The good inquiry for balsam Tolu cannot be met. Cocaine still tends upwards in price. Higher prices are asked for manna. Golden seal is dearer. Makers of seidlitz powder and tartarated soda have raised their quotations. The position of bromides is unchanged. The output of British-made saccharin is increasing. Carbonate of magnesia is dearer, and it is no easy matter to get supplies. Higher prices are quoted for sassafras oil, caraway oil, and cinnamon-leaf oil. A small steady business continues to be done in quinine at unchanged rates. Cod-liver oil is likely to be still higher in price.

### TO OUR READERS.

Contributions are specially invited from any of our readers to these columns. They should deal with topical subjects and news. They must be authenticated for the information of the Editor only. The minimum payment if published is 5s. There is no hard-and-fast rule as to space, but notes of about twenty lines in length are preferred.

